# Effect of Cation Structure in Quinolinium-Based Ionic Liquids on the Solubility in Aromatic Sulfur Compounds or Heptane: Thermodynamic Study on Phase Diagrams

**DOI:** 10.3390/molecules25235687

**Published:** 2020-12-02

**Authors:** Marta Królikowska, Marek Królikowski, Urszula Domańska

**Affiliations:** 1 Department of Physical Chemistry, Faculty of Chemistry, Warsaw University of Technology, Noakowskiego 3, 00-664 Warsaw, Poland; mkrolikowski@ch.pw.edu.pl; 2 Thermodynamics Research Unit, School of Chemical Engineering, University of KwaZulu-Natal, Howard College Campus, King George V Avenue, Durban 4041, South Africa; 3 ŁUKASIEWICZ Research Network—Industrial Chemistry Institute, Rydygiera 8, 01-793 Warsaw, Poland; ula@ch.pw.edu.pl

**Keywords:** desulphurization of fuels, ionic liquids, phase diagrams, NRTL correlation

## Abstract

Experimental and theoretical studies on thermodynamic properties of quinolinium-based ionic liquids (ILs) based on bis(trifluoromethylsulfonyl)imide anion (namely *N*-butyl-quinoloinium bis(trifluoromethylsulfonyl)imide, [BQuin][NTf_2_], *N*-hexylquinoloinium bis(trifluoromethyl-sulfonyl)imide, [HQuin][NTf_2_], and *N*-octylquinoloinium bis(trifluoromethyl-sulfonyl)imide, [OQuin][NTf_2_]) with aromatic sulfur compounds and heptane, as a model compound of fuel were examined in order to assess the applicability of the studied ionic liquids for desulfurization of fuels. With this aim, the temperature-composition phase diagrams of 13 binary mixtures composed of organic sulfur compounds (thiophene, benzothiophene, or 2-methylthiophene) or heptane and ionic liquid (IL) were investigated at ambient pressure. A dynamic method was used to determine the (solid–liquid) equilibrium phase diagrams in binary systems over a wide composition range and temperature range from *T* = 255.15 to 365.15 K up to the fusion temperature of ILs. The immiscibility gap with an upper critical solution temperature (UCST) was observed for each binary system under study. The influence of the alkane chain length of the substituent on the IL cation and of the sulfur compounds (the aromaticity of the solvent) was described. The experimental (solid + liquid) phase equilibrium dataset were successfully correlated using the well-known NRTL equation.

## 1. Introduction

It has been well-known for many years that sulfur compounds are the third, after carbon and hydrogen, most common elements in crude oil, the combustion of which causes the emission of sulfur dioxide [1]. The acid rain, arising from dissolved sulfur compounds in atmosphere and water, cause forest degradation, leading to changes in the ecosystem [1]. It is therefore an important target for many countries to limit the content of sulfur-compounds in the fuel. The process of the desulfurization of fuels is an element of both academic and industrial interest. The existing requirements for diesel fuel range from 10 ppm in Europe, the U.S., and Australia to even above 2000 ppm in economically less developed countries [2]. Restrictions at 10 ppm pose a major technological challenge for science. The currently used method of hydro-desulfurization (HDS) is noneconomic and is ineffective at removing aromatic sulfur compounds (i.e., thiophene, benzothiophene, dibenzo-thiophene and their derivatives), in particular, 4,6-dimethyl-dibenzothiophene, which accounts for more than 55% of the total sulfur content [2]. For decades, new, alternative methods have been proposed such as extractive desulfurization (EDS) [3]. The EDS concept postulates the use of a suitable solvent for the selective separation of sulfur compounds including ionic liquids (ILs) [3,4,5,6,7]. The dominant advantages of EDS compared to HDS are the mild conditions of the process without the use of expensive hydrogen and complex metal catalysts. Nowadays, the concept of using the EDS method after the initial desulfurization using HDS is leading in the literature in terms of profitability. In this case, the conditions of the HDS process may be changed because hydrodesulfurization is aimed at removing only simple sulfur compounds [4]. An economic condition for using EDS is to find a suitable solvent that does not dissolve in fuel, but actively interacts with sulfur compounds. The potential extractants are selected on its polarity. The literature proposes various solutions, among which two groups of compounds dominate: deep eutectic solvents (DES) and ionic liquids (ILs) [3,4,5,6,7].

ILs are an important class of new compounds characterized by stand out extracting properties and eco-friendly (“green”) solvents. ILs reveal low volatility and the possibility of designing their structure, which make them attractive for use in many branches of the chemical industry including extraction processes and environmental clean-up technologies [4,5,6]. ILs are known for high values of selectivity of extraction of aromatic sulfur compounds (for example, thiophene) from aliphatic hydrocarbons (fuels, for example, heptane) in comparison with organic solvents used now on an industrial scale such as NMP or sulfolane [7].

Determining the efficiency of the IL as a solvent in the processes of EDS for fuels is mainly based on experimental measurements of activity coefficients at infinite dilution [8,9,10,11] and on the measurements of phase equilibria in binary- and ternary-systems composed of the IL and heptane, treated as model fuel or/and aromatic sulfur compounds (thiophene, benzothiophene, 2-methylthiophene) [12,13,14,15,16,17]. This is based on an extensive experimental database including the diversity of the cation and anion structure of the IL, which has created many computer simulation methods and the selection of interesting compounds for applications in EDS [7,11].

The (solid + liquid) phase equilibrium (SLE), and the (liquid + liquid) phase equilibrium (LLE) in binary systems composed of quinolinium, or isoquinolinium-based ILs with bis{(trifluoromethyl)-sulfonyl}imide anion as well as their thermophysical properties have been investigated in our laboratory [18,19,20,21,22,23]. So far, the synthesis, thermophysical properties including temperatures and heat effects of phase transitions, the temperature dependence of the density and viscosity of pure ILs as well as the solubility in aromatic hydrocarbons and alcohols have been presented for the following ILs: *N*-butylquinolinium bis{(trifluoromethyl)sulfonyl}imide, [BQuin][NTf_2_] [18], *N*-isobutyl-quinolinium bis{(trifluoromethyl)sulfonyl}imide, [BiQuin][NTf_2_] [19], *N*-hexylquinolinium bis{(trifluoromethyl)sulfonyl}imide, [HQuin][NTf_2_] [20], *N*-hexyliso-quinolinium bis-{(trifluoromethyl)sulfonyl}imide, [HiQuin][NTf_2_] [21], *N*-octylquinolinium bis{(trifluoro-methyl)sulfonyl}imide, [OQuin][NTf_2_] [22], and *N*-octylisoquinolinium bis{(tri-fluoromethyl)-sulfonyl}imide [OiQuin][NTf_2_] [23]. The binary systems of these compounds with aromatic hydrocarbons and alcohols have shown the eutectic systems with immiscibility in the liquid phase with an Upper Critical Solution temperature (UCST). The solubility of these ILs in benzene revealed the lowest immiscibility gap (the highest solubility of benzene in the IL) in comparison with alkylbenzenes (toluene, ethylbenzene, and *n*-propylbenzene). For some of these ILs such as [BQuin][NTf_2_], [OQuin][NTf_2_], and [OiQuin][NTf_2_], the mutual solubility with thiophene were presented earlier [24,25]. In the case of a binary system with thiophene, the similar shape of the SLE curve was reported as for a close-melting substances. However, the difference was observed in the LLE with thiophene, where the immiscibility gap was less steep and the UCST was below the boiling point of the solvent. The UCST was determined experimentally as *T*^UCST^ = 325.6 K (*x*_1_ = 0.0174) and *T*^USCT^ = 355.0 K (*x*_1_ = 0.0227) for [OiQuin][NTf_2_] and [OQuin][NTf_2_], respectively [24,25]. When comparing LLE of the thiophene systems for different quinolinium-based ILs, the immiscibility gap increased with the decrease of an alkyl chain length substituent in the cation of the IL. Changing the cation from quinolinium to isoquinolinum, a lower immiscibility gap was observed due to the better accessibility of the alkyl chain [24,25]. For modeling new technological processes such as extractive distillation or liquid–liquid extraction, the knowledge of the phase equilibria, both SLE, and LLE, and the correlation and prediction of the phase equilibria is very important. The fundamental changes in phase equilibria are connected with different substituents in the cation or anion of the IL This knowledge is important for ILs used as replacement solvents in industry. 

The quinolinium-based ILs were investigated earlier with great extraction potential in the desulfurization of oils (extraction of dibenzothiophene from *n*-dodecane) and in the 1-hexene/*n*-hexane separation problems [24,25]. Therefore, one may expect that the ILs based on the equinolinium or isoquinolinium cation may be considered for industrial applications. Thus far, only a few reports concerning the solubility of these ILs in sulfur-compounds have been published and this was a motivation to undertaking the research presented in this work.

This paper is a continuation of our wide-ranging investigation into the thermodynamics of the phase equilibrium of systems containing ILs. First of all, new experimental solubility data are presented for 13 binary systems. Dynamic method coupled with visual detection was used for the solubility curve determination in the SLE and LLE data. The goal of this work was to assess the suitability of quinolinium or isoquinolinium-based ILs for use in the solvent-extraction of sulfur compounds from liquid fuels. All of the studied ILs had a different core of the cation and different alkyl chain substituted at the cation and hence the impact of the nature of the cation on solubility was demonstrated and discussed. The experimental data were modeled by using the Non-Random Two Liquid model (NRTL).

## 2. Results and Discussion

This work presents the experimental phase equilibrium data for the binary mixtures of ILs with aromatic sulfur compounds and heptane. The experiment was performed using a dynamic method to obtained phase diagrams at a wide temperature and composition range. The molecular interaction and the activity coefficients in binary systems have shown the influence of different factors such as (1) alkyl chain length of substituent in the IL cation; (2) the position of the substituent at the IL cation; and (3) the structure of the aromatic sulfur compound on mutual solubility. 

**Figure 1:**
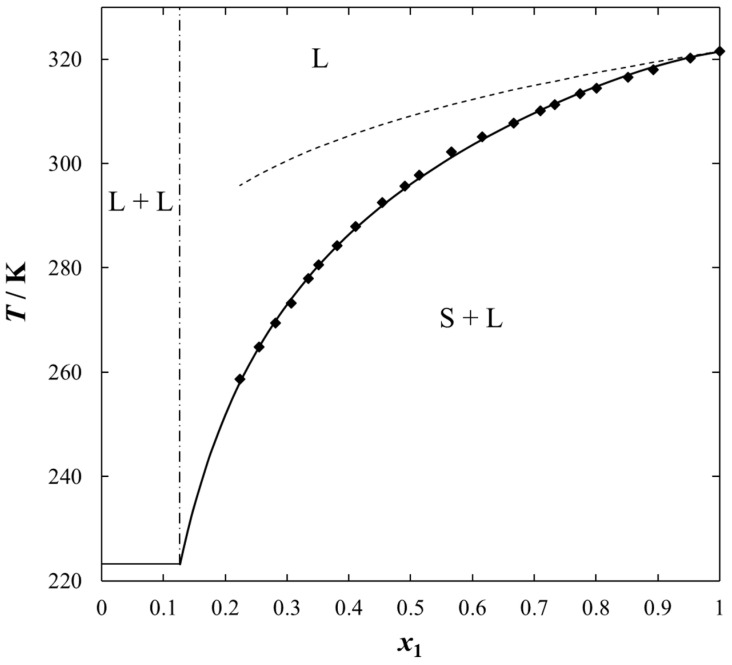
Experimental and calculated (solid + liquid), SLE and (liquid + liquid), LLE phase equilibrium data for {[BiQuin][NTf_2_] (1) + thiophene (2)} binary system. Points show the experimental data; solid line is the correlation of NRTL model with parameters given in Table 5; dashed line is the ideal solubility; dash-dotted line is the experimental binodal curve.

**Figure 2:**
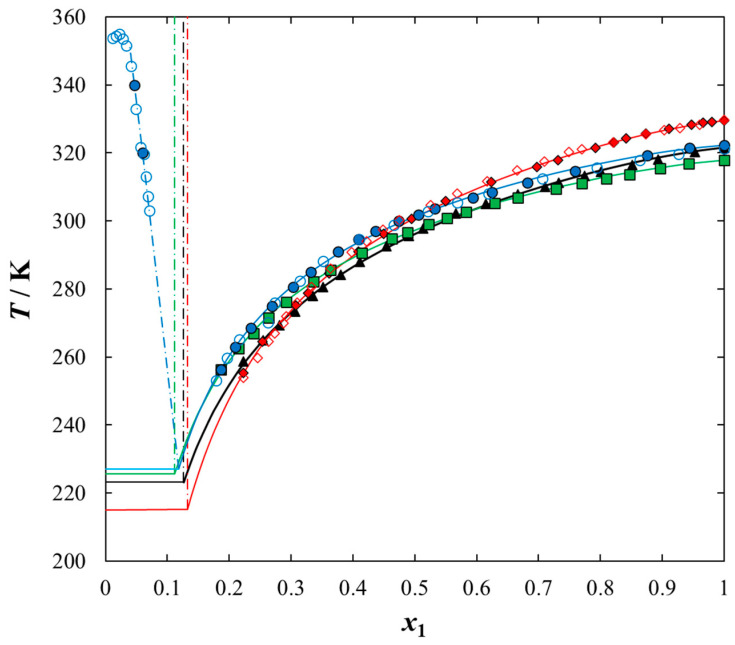
Experimental and calculated (solid + liquid), SLE and (liquid + liquid), LLE phase equilibrium data for {IL (1) + thiophene (2)} binary system: ♦, [BQuin][NTf_2_]; ◊, [BQuin] [NTf_2_] [18]; ▲, [BiQuin][NTf_2_]; ■, [HQuin][NTf_2_]; ●, [OQuin][NTf_2_]; ○, [OQuin][NTf_2_] [22]. Points show the experimental data; solid line is thee correlation of NRTL model with parameters given in Table 5; dash-dotted line is the experimental binodal curve.

**Figure 3:**
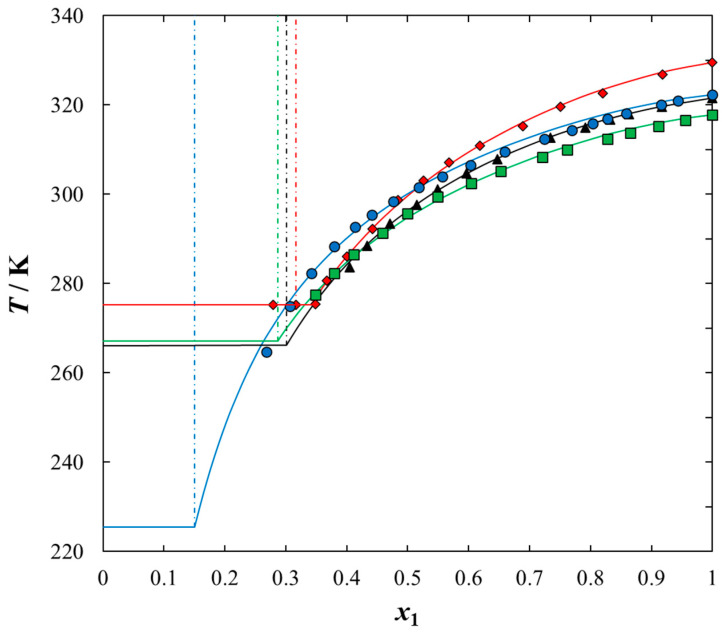
Experimental and calculated (solid + liquid), SLE and (liquid + liquid), LLE phase equilibrium data for {IL (1) + benzothiophene (2)} binary system: ♦, [BQuin][NTf_2_]; ▲, [BiQuin][NTf_2_]; ■, [HQuin][NTf_2_]; ●, [OQuin][NTf_2_]. Points show the experimental data; solid line is the correlation of NRTL model with parameters given in Table 5; dash-dotted line is the experimental binodal curve.

**Figure 4:**
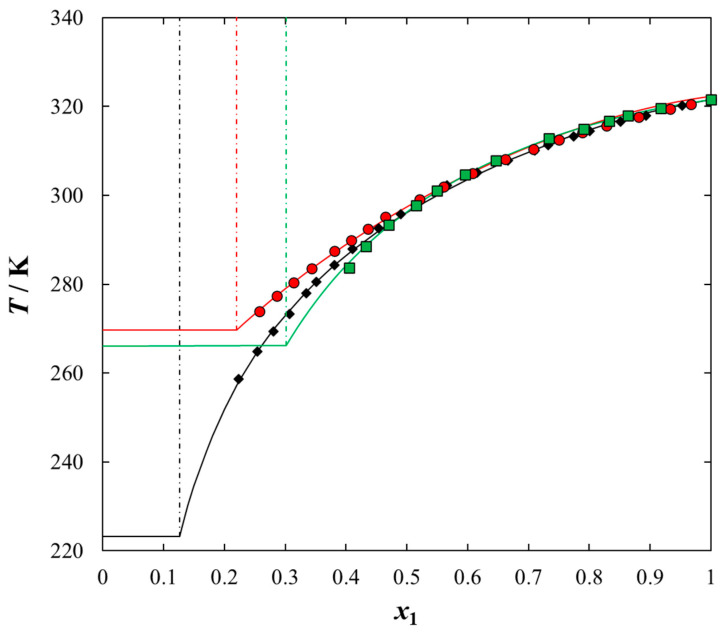
Experimental and calculated (solid + liquid), SLE and (liquid + liquid), LLE phase equilibrium data for {[BiQuin][NTf_2_] (1) + organic sulfur compound (2)} binary system: ♦, thiophene; ●, 2-methylthiophene; ■, benzothiophene. Points are the experimental data; solid line is the correlation of NRTL model with parameters given in Table 5; dash-dotted line is the experimental binodal curve.

**Figure 5:**
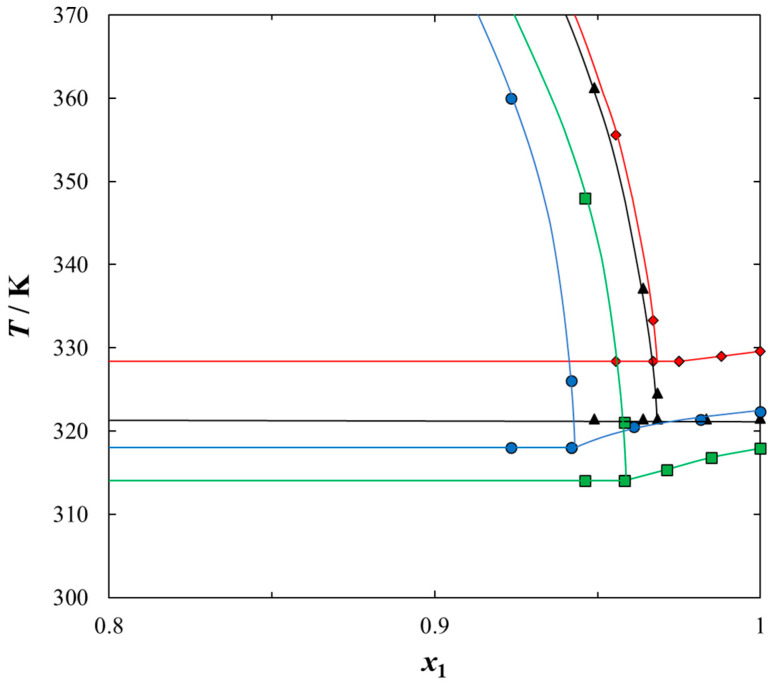
Experimental (solid + liquid), SLE and (liquid + liquid), LLE phase equilibrium data for {IL (1) + heptane (2)} binary system: ♦, [BQuin][NTf_2_]; ▲, [BiQuin][NTf_2_]; ■, [HQuin] [NTf_2_]; ●, [OQuin][NTf_2_]. Solid line is the guide to the eye.

Solubilities of ILs in the sulfur compounds and in heptane, expressed as mole fraction (*x*_1_) of the IL at different equilibrium temperature (*T*) at saturated solution are listed in Table 1, Table 2, Table 3, Table 4 and Table 5. The mathematical description of the SLE data was carried out using the NRTL equation. Table 5 lists the results of fitting the solubility curves by the NRTL model. The resulting SLE phase diagrams, along with the NRTL correlations (solid lines), are graphically presented in Figure 1, Figure 2, Figure 3, Figure 4 and Figure 5. All phase diagrams revealed a large immiscibility gap of LLE with the UCST. The phase diagram for {[BiQuin][NTf_2_] + thiophene} (see Figure 1) is a representative system, where the liquid–liquid immiscibility window is at low mole fraction of the IL (*x*_1_ = 0.13). The solubility of thiophene (2) in the tested ILs was high and the immiscibility gap started from the mole fraction of the IL *x*_1_ = 0.1265 for [BiQuin][NTf_2_], *x*_1_ = 0.1325 for [BQuin][NTf_2_], *x*_1_ = 0.1120 for [HQuin][NTf_2_], and *x*_1_ = 0.0600 for [OQuin][NTf_2_]. The results show the influence of the structure of the IL cation on solubility. The lower immiscibility gap was for the longest alkane substituent chain in the cation, [OQuin][NTf_2_]. The UCST was observed only for [OQuin][NTf_2_] in thiophene at *T* = 358 K in comparison with other quinolinium-based ILs [24]. Unfortunately, due to the temperature limitation of the experimental method and the difficult crystallization, the UCST point and the eutectic points were not determined in the measured systems of this work. When comparing LLE in the binary systems with thiophene for different quinolinium-based ILs, it was observed that the immiscibility gap increased with the decrease in the alkyl chain length of the substituent of the IL cation. As presented in Figure 2, the solubility of thiophene increased in the following order: [BQuin][NTf_2_] < [HQuin][NTf_2_] < [OQuin][NTf_2_]. This trend was previously observed for the solubility of the quinolinium-based ILs in aromatic hydrocarbons [24]. Changing cation from the quinolinium to isoquinolinium-based IL, a lower immiscibility gap was observed due to the better accessibility of the cation in the solution. The solubility of the [BiQuin][NTf_2_] was close to the [OQuin][NTf_2_] (see Figure 2), but the melting temperatures were also very close to each other (see Table 8). The shape of the SLE curves in the measured systems and presented earlier in benzene were similar, as it strictly depends on the melting temperature of the IL [18,19,20,21,22,23]. In the system with benzene, a systematic shift of the eutectic point with an increase in the alkyl chain length of the substituent at the cation toward the lower concentration of the IL was observed [18,19,20,21,22,23]. The same trend was observed for benzothiophene, as is presented in Figure 2. In this case, the immiscibility gap begins from the mole fraction of IL: *x*_1_ = 0.3012 for [BiQuin][NTf_2_], *x*_1_ = 0.3166 for [BQuin][NTf_2_], *x*_1_ = 0.2864 for [HQuin][NTf_2_], and *x*_1_ = 0.1500 for [OQuin][NTf_2_]. The lowest immiscibility gap was observed for [OQuin][NTf_2_]. In the first place, it may be related to the observed melting temperature, but the (CH_2_) hydrophobic group also provides some new effects on mutual interactions of the IL and the solvent.

As expected, the effect of the alkyl chain was observed when introducing the alkyl chain to the aromatic sulfur compound. The solubility of the IL in benzothiophene in comparison with thiophene decreased and the immiscibility gap increased. The wider mole fraction range of the LLE was observed for 2-methylthiophene and benzothiophene (see Figure 4). The immiscibility gap was noted for [BiQuin][NTf_2_] at a mole fraction: *x*_1_ = 0.1265, or *x*_1_ = 0.2196, or *x*_1_ = 0.3012 for thiophene, 2-methylthiophene and benzothiophene, respectively. On the basis of the solubility data, it is evident that the larger immiscibility gap in the system with benzothiophene is additionally the result of the steric hindrance on the intermolecular interactions in the binary system.

In this work, the longer alkyl chain group of the IL cation promoted stronger and favorable interactions between the IL and each solvent tested in this work, which results in a higher solubility (lower immiscibility gap). Thus, the packing effect is probably the most important factor. It has been found that the interaction between X and Y is influenced not only by the packing effects, but also by steric hindrances. As shown in Figure 4, as the length of the alkyl chain in the solvent increases, the incompatibility gap increases. Moreover, the presence of an aromatic ring in the structure of an organic solvent (benzothiophene) significantly reduces the solubility. This is undoubtedly a steric hindrance.

The high solubility of thiophene and other aromatic sulfur compounds in the quinolinium-based ILs (the low immiscibility gaps) confirmed that they may be proper entrainers for the extraction of aromatic sulfur compounds from aliphatic hydrocarbons (fuels). This is additionally explained by the values of activity coefficients of ILs in sulfur compounds (see Table 1, Table 2 and Table 3). All values of activity coefficients were lower than one (*γ*_1_ < 1), which means that the solubility was larger than the ideal solubility (see also Figure 1). 

To use the quinolinium-based ILs as extractants of sulfur compounds from fuels, the solubility of ILs in the model compound, heptane, must be very low. The mutual solubility of quinolinium-based ILs in heptane was determined and the experimental SLE and LLE data are graphically presented in Figure 5. As was shown in the {IL + heptane} systems, there was a wide immiscibility gap due to the weak interaction between the IL and an aliphatic hydrocarbon. There was much stronger interaction between each IL and aromatic sulfur compounds (thiophene, 2-methylthiophene, or benzothiopnehe) compared to that between IL and heptane. Thus, the aromatic sulfur compounds were more soluble in quinolinium-based ILs than aliphatic hydrocarbons. As shown in Figure 5, the solubility of heptane increased with an increase in the alkyl chain length in the IL cation, thus the highest solubility (the lowest immiscibility gap) was observed between heptane and [OQuin][NTf_2_]. This is the impact of the Van der Waals interaction between two alkane chains: an octyl chain as a substituent of the IL cation and an aliphatic chain of heptane. The longer the alkane chain, the stronger the interaction between the IL and a solvent.

Analyzing the possible use of the tested ILs for the separation of the sulfur compounds from the model fuel, the discussion of the solubility of the ILs in aromatic sulfur compounds such as thiophene, 2-methylthiophene, or benzothiophene and in *n*-heptane has to be compared for different ILs. The impact of the cation structure of the IL on solubility depicted for four quinolinium-based ILs shows that [OQuin][NTf_2_] IL is the most promising in comparison with [HQuin][NTf_2_], [BQuin][NTf_2_] and [BiQuin][NTf_2_].

## 3. Materials and Methods

### 3.1. Materials

Chemical structure of the studied ILs, name, abbreviation, molecular mass, and CAS number are presented in Table 6. ILs tested in this work, namely, *N*-butylisoquinoloinium bis(trifluoromethylsulfonyl)imide, [BiQuin][NTf_2_], *N*-butylquinoloinium bis(trifluoromethyl-sulfonyl)imide, [BQuin][NTf_2_], *N*-hexylquinoloinium bis(trifluoromethylsulfonyl)imide, [HQuin][NTf_2_], and *N*-octylquinoloinium bis(trifluoromethylsulfonyl)imide, [OQuin][NTf_2_] were synthesized in our laboratory. The detailed description of the synthesis procedure and analysis have been previously published [18,19,20,21,22,23]. Briefly, for the synthesis of 1-alkylquinolinium bromide, or 1-alkylisoquinolinium bromide, a 750 cm^3^ flask in an oil bath, equipped with a magnetic stirrer, condenser, and drying tube was used. To a solution of quinoline or isoquinoline in 200 cm^3^ acetonitrile, 1-alkylbromide was added. The stirred mixture was heated to reflux for 24 h. Afterward, the solvent was removed in vacuum. The product was diluted with 200 cm^3^ of water, and extracted with diethyl ether ten times at 50 cm^3^ each until the latter remained colorless. Water was removed in vacuum. The product was further crystallized from ethanol and dried under vacuum at *T* = 353.15 K for 12 h.

For the synthesis, a 250 cm^3^ flask, equipped with a magnetic stirrer and condenser was used. To a solution of 1-alkylquinolinium bromide or 1-alkylisoquinolinium bromide in 500 cm^3^ water, lithium bis{(trifluoromethyl)sulfonyl}imide was added. The solution became lighter in color and a second heavier phase was formed. The mixture was stirred for 24 h, afterward, the phases were separated. The heavier phase was diluted with 50 cm^3^ dichloromethane and extracted with distilled water until the aqueous phase gave a negative response on the addition of AgNO_3_. Dichloromethane was removed in vacuum, and the product was dried under vacuum at *T* = 353.15 K for 12 h. The list of reagents used in the synthesis of ILs is provided in Table S1 in the Supplementary Materials (SM). 

Before the measurements, the ILs were degassed and dried in vacuum to diminish the volatile chemicals and water content. Purification was provided at a very low pressure of about *p* = 5 × 10^−3^ Pa, obtained by a vacuum pump vacuum drying oven (Binder, model VD 23, Vacuubrand GMBH+ CO KG, Wertheim, Germany) at temperature *T* = 363.15 K for a minimum 24 h. Afterward, the resulting water content was estimated by coulometric titration (Karl–Fischer method) using a KF Trace Titroline (Metrohm, 716 DMS Titrino, Weilheim, Germany). In this analysis, the sample of IL was dissolved in dry methanol and titrated with steps of 2.5 μL. After three titrations, the average water content in the tested sample was determined and the results are listed in Table 7. All solvents were stored over a freshly activated molecular sieve of type 0.4 nm (Union Carbide, Houston, TX, USA).

The thermophysical characterization of pure ILs using the differential scanning calorimetry (DSC) technique was presented in our earlier work [18,19,20,22]. To show the results, the fundamental data of glass transition temperature and melting temperature as well as the corresponding heat effects of these transitions are presented in Table 8.

### 3.2. Karl–Fischer Analysis

The water content was analyzed by the Karl–Fischer titration technique (method TitroLine KF). The sample of IL was dissolved in methanol and titrated with steps of 2.5 μL. The results obtained for each IL are presented in Table 7. The error on the water content was (10 ppm for the 3 mL of injected IL. The water content below 290 ppm described the purity of the ILs. Usually, the large water content is changing the density and viscosity of the ILs and has an influence on the phase equilibria. The solubility of different compounds will change for water-containing solvents.

### 3.3. Phase Equilibria Apparatus and Measurements

(Solid + liquid) and (liquid + liquid) phase equilibria, SLE and LLE, were determined using the well-known dynamic (synthetic) method [20]. The mixtures of the IL and the solvent were prepared by weighing the pure components on Mettler Toledo AB204-S balance (Greifensee, Switzerland) with an uncertainty of 1 × 10^−4^ g. The Pyrex glass cell with a well-known mole fraction (*x*_1_) solution was immersed in a thermostatic bath. The sample was heated very slowly (less than 2 K·h^−1^ near the equilibrium temperature) with continuous and vigorous stirring inside the cell. The solid phase disappearance temperatures, detected visually, (the equilibrium temperature of last crystal disappearance in the case of SLE measurements), or two phase disappearance (in the case of LLE measurements) during increasing temperature were measured with an electronic thermometer P550 (DOSTMANN Electronic GmbH, Wertheim, Germany). The uncertainty of the temperature measurement was 0.1 K. The experiments were carried out over a wide range of the IL mole fractions (*x*_1_) at a temperature range up to *T* = 365 K at pressure, *p* = 0.1 MPa.

### 3.4. Correlation with NRTL Equation

Little is known about the SLE of the ILs. The quinolinium-based ILs revealed high fusion temperature, thus some solubilities in hydrocarbons and alcohols have already been published [24,25]. The SLE in the binary system (components *i*,*j*) with the pure solid phase of component *i* and the saturated solution of *i* as the second phase, is described by below equation [26]:

(1) $$\ln x_{i}=-\frac{\Delta_{fus}H_{i}}{RT}\left(1-\frac{T}{T_{fus,i}}\right)-\ln\gamma_{i}\left(x_{i},\;T,\;\Delta g_{ij},\;\Delta g_{ji}\right)$$

where the $\Delta_{fus}H_{i}$ stands for the enthalpy of fusion; *R* for the ideal gas constant; $T_{fus,i}$ is the normal temperature of fusion; and $\gamma_{i}$ is an activity coefficient of solute *i* in the liquid phase. When the solution is assumed as ideal ($\gamma_{i}=1$), the SLE phase diagram can be modeled based only on the melting properties of the pure components. Ideal solubility is represented by the dashed line in Figure 1. However, if the system deviates from ideal behavior, the activity coefficients of components in the liquid phase can be calculated using well-developed *g*^E^ models or equations of state. For the systems from this work, the NRTL model was chosen. The activity coefficient, $\gamma_{i}$, depends on $x_{i}$, *T*, and some parameters $\Delta g_{ij},\;\Delta g_{ij}$, provided by the NRTL model. 

**Figure 6:**
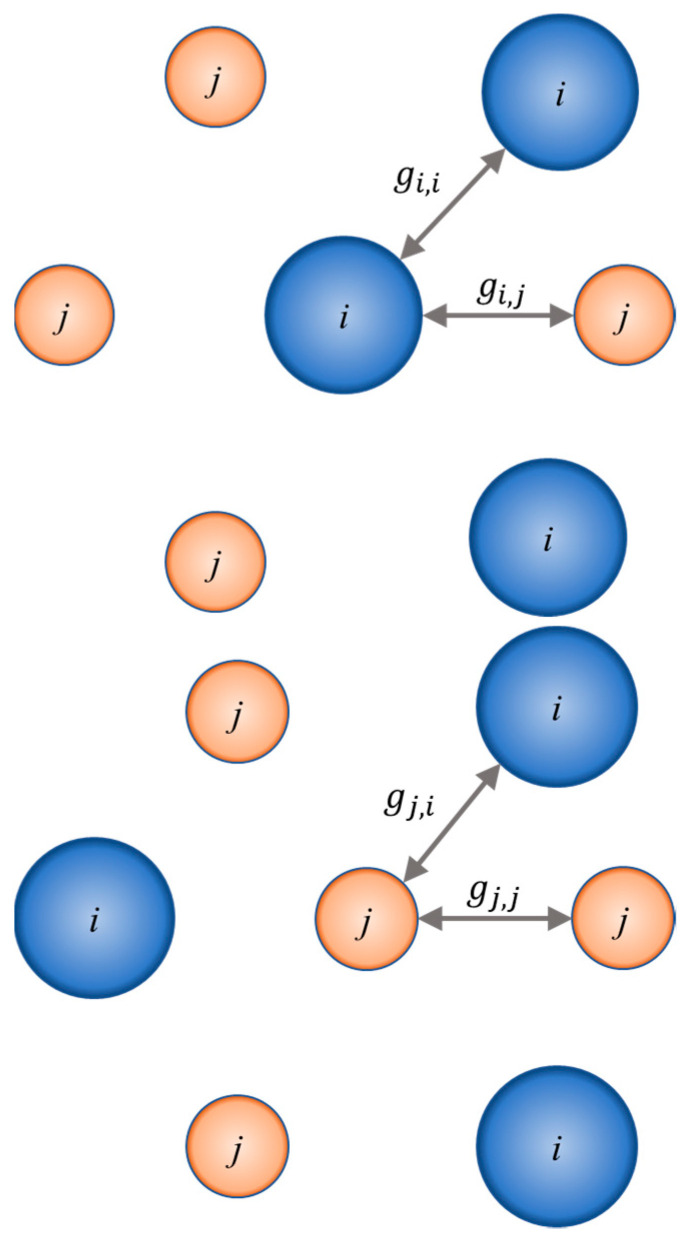
Two types of molecular clusters or cells of binary liquid mixtures. Each molecule of type *i* or of type *j* is regarded as being surrounded by molecules of both types in proportions determined by the Gibbs energies of interaction, *g_jj_*, which are indicated in the diagram.

The NRTL equation of the excess Gibbs energy is a two-cell theory represented in Figure 6. It is assumed that the liquid has a structure made up of cells of molecules of types *i* and *j* in a binary mixture, each surrounded by assortments of the same molecules, with each of the surrounding molecules in turn surrounded in a similar manner, and so on. Gibbs energies of interaction between molecules are identified by $g_{ij}$, where subscript *i* refers to the central molecule.

By fixing the composition $x_{i}$ and parameters $\Delta g_{ij},\;\Delta g_{ji}$ in Equation (1), one can calculate equilibrium temperature *T*.

By minimalization of the following objective function:

(2) $$F\left(\Delta g_{ij},\;\Delta g_{ji}\right)=\underset{i}{\sum }\left\{T_{i}^{exp}-T_{i}^{cal}\left(\Delta g_{ij},\;\Delta g_{ji}\right)\right\}$$

the model parameters may be obtained. 

The root-mean-square deviation of temperature, $\sigma_{T}$, has been calculated according to the following definition:

(3) $$\sigma_{T}={\left\{\overset{n}{\underset{i=1}{\sum }}\frac{{\left(T_{i}^{exp}-T_{i}^{cal}\left(\Delta g_{ij},\;\Delta g_{ji}\right)\right)}^{2}}{n-2}\right\}}^{\frac{1}{2}}$$

where *n* is the number of experimental points (including the melting point), and two is the number of adjustable parameters.

The activity coefficient in the NRTL model is given by

(4) $$\ln\gamma_{i}=x_{j}^{2}\left[\tau_{21}{\left(\frac{G_{ji}}{x_{i}+x_{j}G_{ji}}\right)}^{2}+\frac{\tau_{ij}G_{ij}}{{\left(x_{j}+x_{i}G_{ij}\right)}^{2}}\right]$$

where

(5) $$G_{ij}=\exp\left(-\alpha_{ij}\tau_{ij}\right)\;\;\;\;\;\;\;\;\;\;\;G_{ji}=\exp\left(-\alpha_{ij}\tau_{ji}\right)$$

(6) $$\;\tau_{ij}=\frac{g_{ij}-g_{jj}}{RT}\;\;\;\;\;\;\;\;\;\;\;\;\;\;\tau_{ji}=\frac{g_{ji}-g_{ii}}{RT}\;$$

The NRTL model contains three parameters: $\Delta g_{ij}=g_{ij}-g_{jj}$, $\Delta g_{ji}=g_{ji}-g_{ii}$, and $\alpha_{ij}$. The third parameter $\alpha_{ij}$ is related to the nonrandomness in the mixtures and varies from 0.20 to 0.78. 

## 4. Conclusions and Future Perspectives

The experimental data of solubility of quinolinium-based ILs, [BiQuin][NTf_2_], [BQuin][NTf_2_], [HQuin][NTf_2_], and [OQuin][NTf_2_] in aromatic sulfur compounds (i.e., thiophene, 2-methylthiophene and benzothiophene as well as in heptane) at a wide temperature and composition range at ambient pressure were presented. As a result, one can conclude that the mutual solubility in the investigated systems depends on many factors including the length of the alcohol chain in the ionic liquid cation, the length of the alkyl chain in the aromatic sulfur compound, and the aromaticity of the solvent. In particular, the longer alkyl chain of the quinolinium cation and the shorter alkyl chain of the solvent promotes higher solubility. It was observed that the quinolinium–based ILs tested in this work exhibited high solubility in aromatic sulfur compounds and a very low solubility in heptane. The experiment shows that with an increase in the alkyl chain length in the cation substituent, the solubility increases, thus the lowest immiscibility gap was observed in the systems with [OQuin][NTf_2_] and the highest for [BQuin][NTf_2_]. Moreover, the change in the cation from quinolinium to isoquinolinium caused a slight increase in the solubility of each of the tested solvents. Finally, it should be emphasized that solubility of the ILs larger than the ideal one was observed in all sulfur compounds. The large differences between the solubility in aromatic sulfur compounds and heptane allows us to state that the ILs tested in this research may be good extractants of sulfur compounds from liquid fuels. Experimental SLE data were correlated with the NRTL model with a root mean square deviation lower than one.

## Figures and Tables

**Table 1:** Experimental (solid + liquid) phase equilibrium data, SLE, for {IL (1) + thiophene (2)} binary systems at pressure *p* = 0.1 MPa: *x*_1_ is the ionic liquid mole fraction; *T*^SLE^ is the equilibrium temperature; *γ*_1_ is the activity coefficient of the IL obtained with the NRTL equation ^a,b^.

*x*_1_	*T*^SLE^/K	*γ*_1_	*x*_1_	*T*^SLE^/K	*γ*_1_
**[BiQuin][NTf_2_] (1) + thiophene (2)**					
1.0000	321.5	1.00	0.5139	297.8	0.48
0.9527	320.2	0.99	0.4904	295.7	0.44
0.8930	318.0	0.95	0.4541	292.5	0.38
0.8515	316.5	0.92	0.4106	287.9	0.32
0.8008	314.5	0.87	0.3808	284.2	0.27
0.7746	313.3	0.83	0.3514	280.5	0.23
0.7328	311.3	0.78	0.3347	277.9	0.20
0.7105	310.1	0.75	0.3066	273.3	0.16
0.6664	307.8	0.69	0.2808	269.4	0.13
0.6153	305.1	0.62	0.2543	264.8	0.10
0.5665	302.2	0.55	0.2231	258.7	0.06
**[BQuin][NTf_2_] (1) + thiophene (2)**					
1.0000	329.6	1.00	0.6235	311.4	0.62
0.9807	329.2	1.00	0.5497	305.8	0.51
0.9664	328.8	0.99	0.4948	300.6	0.42
0.9476	328.3	0.99	0.4499	296.3	0.35
0.9106	327.0	0.97	0.4094	291.0	0.29
0.8736	325.6	0.94	0.3621	284.5	0.22
0.8415	324.2	0.91	0.3279	278.8	0.16
0.8216	323.1	0.89	0.3080	275.2	0.14
0.7919	321.5	0.85	0.2542	264.5	0.07
0.7309	317.9	0.78	0.2226	255.2	0.04
0.6972	316.0	0.73			
**[HQuin][NTf_2_] (1) + thiophene (2)**					
1.0000	317.9	1.00	0.5226	299.0	0.42
0.9431	316.8	0.98	0.4878	296.5	0.36
0.8968	315.5	0.95	0.4625	294.8	0.32
0.8472	313.7	0.90	0.4144	290.7	0.25
0.8091	312.4	0.85	0.3633	285.6	0.18
0.7695	311.1	0.80	0.3367	282.2	0.15
0.7284	309.4	0.74	0.2917	276.1	0.09
0.6662	306.8	0.64	0.2638	271.5	0.07
0.6292	305.2	0.58	0.2398	267.0	0.05
0.5830	302.7	0.51	0.2146	262.5	0.03
0.5523	300.8	0.46	0.1860	256.3	0.02
**[OQuin][NTf_2_] (1) + thiophene (2)**					
1.0000	322.3	1.00	0.4361	297.1	0.29
0.9436	321.4	0.98	0.4093	294.6	0.25
0.8762	319.3	0.93	0.3762	291.0	0.20
0.7590	314.7	0.79	0.3319	285.0	0.14
0.6824	311.3	0.68	0.3028	280.7	0.10
0.6244	308.4	0.59	0.2694	275.0	0.07
0.5934	306.8	0.54	0.2345	268.5	0.04
0.5319	303.5	0.44	0.2093	263.0	0.02
0.5061	301.9	0.40	0.1861	256.3	0.01
0.4734	300.0	0.35			

^a^ Standard uncertainties *u* are as follows: *u*(*x*_1_) = 0.0001 and *u*(*T*) = 0.1 K; *u*(*P*) = 3 kPa. ^b^ NRTL parameters are tabulated in Table 5.

**Table 2:** Experimental (solid + liquid) phase equilibrium data, SLE, for {IL (1) + benzothiophene (2)} binary systems at pressure *p* = 0.1 MPa: *x*_1_ is the ionic liquid mole fraction; *T*^SLE^ is the equilibrium temperature; *γ*_1_ is thee activity coefficient of the IL obtained with the NRTL equation ^a,b^.

*x*_1_	*T*^SLE^/K	*γ*_1_	*x*_1_	*T*^SLE^/K	*γ*_1_
**[BiQuin][NTf_2_] (1) + benzothiophene (2)**					
1.0000	321.5	1.00	0.5960	304.7	0.64
0.9175	319.6	0.98	0.5495	301.1	0.56
0.8633	317.9	0.96	0.5153	297.7	0.49
0.8325	316.8	0.94	0.4704	293.4	0.40
0.7910	315.0	0.90	0.4332	288.6	0.33
0.7337	312.8	0.84	0.4052	283.6	0.27
0.6469	307.9	0.72			
**[BQuin][NTf_2_] (1) + benzothiophene (2)**					
1.0000	329.6	1.00	0.4839	298.7	0.37
0.9185	326.8	0.98	0.4419	292.3	0.29
0.8200	322.7	0.90	0.4002	286.0	0.22
0.7509	319.6	0.82	0.3675	280.7	0.16
0.6885	315.3	0.73	0.3481	275.4	0.13
0.6181	310.9	0.62	0.3166	275.2	
0.5674	307.1	0.53	0.2786	275.2	
0.5261	303.0	0.45			
**[HQuin][NTf_2_] (1) + benzothiophene (2)**					
1.0000	317.9	1.00	0.6037	302.4	0.48
0.9559	316.7	0.99	0.5493	299.5	0.39
0.9112	315.3	0.96	0.4990	295.8	0.30
0.8650	313.7	0.91	0.4586	291.4	0.24
0.8277	312.4	0.86	0.4112	286.5	0.17
0.7619	310.0	0.76	0.3797	282.4	0.13
0.7205	308.3	0.69	0.3481	277.5	0.09
0.6521	305.1	0.57			
**[OQuin][NTf_2_] (1) + benzothiophene (2)**					
1.0000	322.3	1.00	0.5573	304.0	0.45
0.9439	320.9	0.99	0.5180	301.6	0.38
0.9160	320.0	0.97	0.4763	298.4	0.31
0.8591	318.1	0.92	0.4415	295.4	0.25
0.8276	316.9	0.88	0.4137	292.7	0.21
0.8039	315.8	0.85	0.3790	288.3	0.15
0.7693	314.3	0.80	0.3418	282.3	0.10
0.7239	312.4	0.74	0.3069	275.0	0.07
0.6591	309.5	0.63	0.2683	264.8	0.03
0.6028	306.5	0.53			

^a^ Standard uncertainties *u* are as follows: *u*(*x*_1_) = 0.0001 and *u*(*T*) = 0.1 K; *u*(*P*) = 3 kPa. ^b^ NRTL parameters are tabulated in Table 5.

**Table 3:** Experimental (solid + liquid) phase equilibrium data, SLE, for {IL (1) + 2-methylthiophene (2)} binary systems at pressure *p* = 0.1 MPa: *x*_1_ is the ionic liquid mole fraction; *T*^SLE^ is the equilibrium temperature; *γ*_1_ is the activity coefficient of the IL obtained with the NRTL equation ^a,b^.

*x*_1_	*T*^SLE^/K	*γ*_1_	*x*_1_	*T*^SLE^/K	*γ*_1_
[BiQuin][NTf_2_] (1) + 2-methylthiophene (2)					
1.0000	321.5	1.00	0.5601	302.0	0.56
0.9665	320.5	1.00	0.5204	299.1	0.50
0.9334	319.4	0.99	0.4646	295.1	0.43
0.8815	317.7	0.96	0.4359	292.5	0.39
0.8280	315.6	0.91	0.4083	290.0	0.35
0.7882	314.1	0.87	0.3813	287.4	0.32
0.7505	312.5	0.82	0.3434	283.6	0.28
0.7082	310.5	0.77	0.3135	280.4	0.24
0.6620	308.2	0.71	0.2865	277.4	0.22
0.6081	305.0	0.63	0.2576	274.0	0.19

^a^ Standard uncertainties *u* are as follows: *u*(*x*_1_) = 0.0001 and *u*(*T*) = 0.1 K; *u*(*P*) = 3 kPa. ^b^ NRTL parameters are tabulated in Table 5.

**Table 4:** Experimental (solid + liquid) phase equilibrium data, SLE, for {IL (1) + heptane (2)} binary systems at pressure *p* = 0.1 MPa: *x*_1_ is the ionic liquid mole fraction; *T*^SLE^ is the equilibrium temperature ^a,b^.

*x*_1_	*T*^SLE^/K	*x*_1_	*T*^SLE^/K	*T*^LLE^/K
**[BiQuin][NTf_2_] (1) + heptane (2)**				
1.0000	321.51	0.9684	321.49	324.54
0.9833	321.49	0.9640	321.49	337.15
		0.9490	321.49	361.29
**[BQuin][NTf_2_] (1) + heptane (2)**				
1.000	329.6	0.967	328.4	333.3
0.988	329.0	0.956	328.4	355.6
0.975	328.4	0.967	328.4	333.3
**[HQuin][NTf_2_] (1) + heptane (2)**				
1.000	317.9	0.958	314.0	321.3
0.985	316.8	0.946	314.0	348.0
0.971	315.3			
**[OQuin][NTf_2_] (1) + heptane (2)**				
1.000	322.3	0.942	318.0	326.0
0.982	321.4	0.923	318.0	360.1
0.961	320.5			

^a^ Standard uncertainties *u* are as follows: *u*(*x*_1_) = 0.0001 and *u*(*T*) = 0.1 K; *u*(*P*) = 3 kPa. ^b^ NRTL parameters are tabulated in Table 5.

**Table 5:** The NRTL fitting parameters, obtained by the correlation of the SLE data for {IL (1) + organic sulfur compound or heptane (2)} binary systems.

System	NRTL Parameters			RMSD
	$g_{12}-g_{11}/J\cdot {\mathrm{mol}}^{-1}$	$g_{21}-g_{22}/J\cdot {\mathrm{mol}}^{-1}$	$\alpha_{12}=\alpha_{21}$	$\sigma_{T}/K$
[BiQuin][NTf_2_] (1) + thiophene (2)	−4591.78	−2579.41	0.78	0.64
[BQuin][NTf_2_] (1) + thiophene (2)	−5915.62	−2776.95	0.71	0.45
[HQuin][NTf_2_] (1) + thiophene (2)	−5797.59	−3159.81	0.62	0.56
[OQuin][NTf_2_] (1) + thiophene (2)	−6318.13	−3125.06	0.62	0.69
[BiQuin][NTf_2_] (1) + benzothiophene (2)	−8888.07	−2032.34	0.46	0.55
[BQuin][NTf_2_] (1) + thiophene (2)	−11,775.80	−2937.43	0.45	0.78
[HQuin][NTf_2_] (1) + thiophene (2)	−11,068.53	−3916.45	0.45	0.80
[OQuin][NTf_2_] (1) + thiophene (2)	−12,273.10	−3527.76	0.45	1.05
[BiQuin][NTf_2_] (1) + 2-methylthiophene (2)	−2000.44	−4000.62	0.20	0.85

**Table 6:** The list of ionic liquids (ILs) under study; name, abbreviation, molecular mass, and CAS number.

Structure	Name, Abbreviation, CAS No.
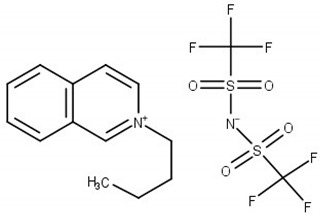	*N*-butylisoquinolinium bis(trifluoromethylsulfonyl)imide, [BiQuin][NTf_2_] *M* = 466.42 g·mol^−1^ CAS No. 957763-47-2
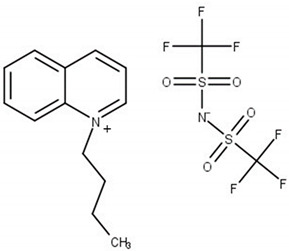	*N*-butylquinolinium bis(trifluoromethylsulfonyl)imide, [BQuin][NTf_2_] *M* = 466.42 g·mol^−1^ CAS No. 1289382-58-6
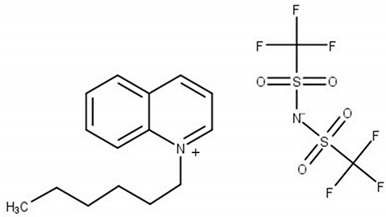	*N*-hexylquinolinium bis(trifluoromethylsulfonyl)imide, [HQuin][NTf_2_] *M* = 494.47 g·mol^−1^ CAS No. 1263302-30-2
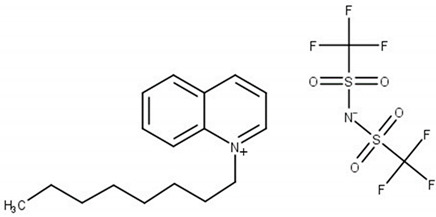	*N*-octylquinolinium bis(trifluoromethylsulfonyl)imide, [OQuin][NTf_2_] *M* = 522.52 g·mol^−1^ CAS No. 868671-34-5

**Table 7:** Specifications of the chemical samples. ^a^

Sample	Source	Initial Mole Fraction Purity (wt%)	Purification Method	Final Mole Fraction Purity (wt%)	Analysis Method	Water Content/ppm
[BiQuin][NTf_2_]	own synthesis	-	crystallization, vacuum heating	97.0	Karl–Fischer, ^1^H-NMR	290
[BQuin][NTf_2_]	own synthesis	-	crystallization, vacuum heating	97.0	Karl–Fischer, ^1^H-NMR	280
[HQuin][NTf_2_]	own synthesis	-	crystallization, vacuum heating	97.0	Karl–Fischer, ^1^H-NMR	280
[OQuin][NTf_2_]	own synthesis	-	crystallization, vacuum heating	97.0	Karl–Fischer, ^1^H-NMR	250
heptane	Sigma-Aldrich ^b^	99.0	-	99.0	-	50
thiophene	Sigma-Aldrich ^b^	99.0	-	99.0	-	70
2-methylthiophene	Sigma-Aldrich ^b^	98.0	-	98.0	-	110
benzothiophene	Sigma-Aldrich ^b^	98.0	-	98.0	-	150

^a^ All solvents were stored on freshly activated molecular sieves; ^b^ Sigma-Aldrich, Poznań, Poland.

**Table 8:** Thermal properties of the investigated ionic liquids: glass transition temperature (*T*_g,1_) and heat capacity change at glass transition temperature (Δ*C*_p(g),1_), melting temperature (*T*_fus_), and enthalpy (Δ_fus_*H*) ^a^.

Ionic Liquid	*T*_g,1_/K	Δ*C*_p(g),1_/J mol^−1^ K^−1^	*T*_fus_/K	Δ_fus_*H*/kJ mol^−1^
[BiQuin][NTf_2_] [19]	217.9	332.4	321.0	46.13
[BQuin][NTf_2_] [18]	199.2	105.9	329.1	44.14
[HQuin][NTf_2_] [20]	219.3	317.7	317.2	63.54
[OQuin][NTf_2_] [22]	219.4	423	321.3	62.91

^a^ standard uncertainties *u* are as follows: *u*(*T*_g,1_) = 0.1 K; *u*(Δ*C*_p(g),1_) = 5 J mol^−1^ K^−1^, *u*(*T*_fus_) = 0.1 K; *u*(Δ_fus_*H*) = 0.5 kJ mol^−1^.

## Supplementary Materials

The following are available online, Table S1: The list of chemicals used for synthesis of ILs.
