# Supplementary material for: Effect of Cation Structure in Quinolinium-Based Ionic Liquids on the Solubility in Aromatic Sulfur Compounds or Heptane: Thermodynamic Study on Phase Diagrams
Source: Molecules. 2020 Dec 2;25(23):5687. doi: 10.3390/molecules25235687 (PMC7731319; doi:10.3390/molecules25235687)
Supplement: Supplementary file 1 [file molecules-25-05687-s001.pdf]

Supplementary Materials

Effect of Cation Structure in Quinolinium-Based Ionic Liquids on the Solubility in Aromatic Sulfur Compounds or Heptane: Thermodynamic Study on Phase Diagrams

Marta Królikowska [1,2,*], Marek Królikowski [1,2] and Urszula Domańska [2,3]

[1] Department of Physical Chemistry, Faculty of Chemistry, Warsaw University of Technology, Noakowskiego 3, 00-664 Warsaw, Poland; mkrolikowski@ch.pw.edu.pl

[2] Thermodynamics Research Unit, School of Chemical Engineering, University of KwaZulu-Natal, Howard College Campus, King George V Avenue, Durban 4041, South Africa; ula@ch.pw.edu.pl

[3] ŁUKASIEWICZ Research Network—Industrial Chemistry Institute, Rydygiera 8, 01-793 Warsaw, Poland

* Correspondence: mlaskowska@ch.pw.edu.pl

Table S1. The list of chemicals used for synthesis of ILs.

Name	CAS No.	Supplier	Purity (%)
Quinoline	91-22-5	Sigma Aldrich	98.0
Isoquinoline	119-65-3	Sigma Aldrich	97.0
1-Bromobutane	109-65-9	Sigma Aldrich	98.0
1-Bromohexane	111-25-1	Sigma Aldrich	98.0
1-Bromooctane	111-83-1	Sigma Aldrich	99.0
lithium bis{(trifluoromethyl)sulfonyl}imide	90076-65-6	Sigma Aldrich	99.0
Acetonitrile	75-05-8	Avantor	99.9
Diethyl ether	60-29-7	Avantor	99.0
Ethanol	64-17-5	Avantor	99.8
Dichloromethane	75-09-2	Avantor	99.5
Silver nitrate	7761-88-8	Sigma Aldrich	99.9
Water (doubly distilled and degassed)	7732-18-5	Own source	-
